# From Challenge to Opportunity: Virtual Qualitative Research During COVID-19 and Beyond

**DOI:** 10.1177/16094069221105075

**Published:** 2022-06-04

**Authors:** Sam Keen, Martha Lomeli-Rodriguez, Helene Joffe

**Affiliations:** Research Department of Clinical, Educational & Health Psychology, 4919 University College London, London

**Keywords:** virtual research, video interviewing, COVID-19, qualitative methodology, grid elaboration method, diversity and inclusion

## Abstract

COVID-19 has required researchers to adapt methodologies for remote data collection. While virtual interviewing has traditionally received limited attention in the qualitative literature, recent adaptations to the pandemic have prompted increased discussion and adoption. Yet, current discussion has focussed on practical and ethical concerns and retained a tone of compromise, of coping in a crisis. This paper extends the nascent conversations begun prior to the pandemic to consider the wider methodological implications of video-call interviews. Beyond the short-term, practical challenges of the pandemic, these adaptations demonstrate scope for longer-term, beneficial digitalisation of both traditional and emergent interview methods. Updating traditional interview methods digitally has demonstrated how conversion to video interviewing proves beneficial in its own right. Virtual focus-group-based research during COVID-19, for example, accessed marginalised populations and elicited notable rapport and rich data, uniting people in synchronous conversation across many environments. Moreover, emergent interview methods such as the Grid Elaboration Method (a specialised free-associative method) demonstrated further digitalised enhancements, including effective online recruitment with flexible scheduling, virtual interactions with significant rapport, and valuable recording and transcription functions. This paper looks beyond the pandemic to future research contexts where such forms of virtual interviewing may confer unique advantages: supporting researcher and participant populations with mobility challenges; enhancing international research where researcher presence or travel may be problematic. When opportunities for traditional face-to-face methods return, the opportunity for virtual innovation should not be overlooked.

## Introduction

The COVID-19 pandemic disrupted our everyday lives (Teti et al., 2020). Beyond working, socialising, holidaying, education and healthcare, it has also disrupted research methods (Rahman et al., 2021): lockdowns and social distancing prevented face-to-face data collection (Lobe et al., 2020). Multiple authors have described resultant technological adaptations and their practical and ethical considerations (Adom et al., 2020; Hall et al., 2021; Lathen & Laestadius, 2021; Topping et al., 2021). However, there has been limited comment on longer-term methodological implications of these adaptations (Roberts et al., 2021). This paper reflects on the potential of virtual qualitative research. It augments the literature by arguing for advantages of remote data collection across traditional and emergent research methods, even as face-to-face opportunities return. This argument seeks to shift the conversation away from ‘coping with’ virtual research when crises require it, towards embracing it as a valid and valuable avenue for methodological innovation.

### Remote Research Methods

#### Video interviewing

Interviewing is the most prevalent mode of data collection in qualitative research (Creswell & Poth, 2016). While remote methods have long been used in quantitative survey research, they have seen limited employment, and even less methodological discussion, in qualitative literature (Roberts et al., 2021). Qualitative methodologists historically recommended face-to-face contact for qualitative interviewing (Gillham, 2005; Rubin & Rubin, 2011), cautioning that remote meeting may impede rapport, engender participant fatigue and restrict depth of interaction (Irvine et al., 2013). Widely cited textbooks on qualitative interviewing disregard distanced interviewing as sub-optimal (Rubin & Rubin, 2011).

However, as video-call platforms grow more advanced and widely used, methodological discussion of video interviewing has emerged in qualitative research (Deakin & Wakefield, 2013). This includes nascent observations of some restrictions – geographical, financial, social – of in-person interviewing (Janghorban et al., 2014) and recognition that video calling offers widened reach (Hooley et al., 2012) and comparable visual cues to face-to-face encounters (Sullivan, 2012). Traditional qualitative methods such as focus groups are increasingly being conducted online (Lobe et al., 2020). Yet, there is still limited exploration of video interviewing in the literature (Chandratre & Soman, 2020; Roberts et al., 2021), and most discussions frame it as a methodological compromise (Deakin & Wakefield, 2013; Lobe et al., 2020).

### Virtual Research in COVID-19

The pandemic prompted global uptake of remote data collection. Recent qualitative research features telephone interviewing (Adom et al., 2020; Hall et al., 2021), Email interviewing (Amri et al., 2021), instant-messaging interviewing (Hinkes, 2020; Woodward et al., 2020) and video interviewing (Dodds & Hess, 2020). Synchronous video interviewing, where participants and researchers interact in real time, has drawn particular interest (Lobe et al., 2020; Roberts et al., 2021; Teti et al., 2020), given its greater scope for emulating natural conversation (Nehls et al., 2015) and establishing rapport above other virtual approaches (Archibald et al., 2019; Deakin & Wakefield, 2013; Sy et al., 2020). Our personal and professional interactions are increasingly digital, and COVID-19 has accelerated digitalisation of qualitative interview practices. By digitalisation we mean the transposition of in-person interviewing to virtual formats.

However, most recent discussions of video interviewing have focussed on its practical, technical and ethical protocols and implications. Reflections have included practical strengths and weaknesses of each video-call platform; logistical considerations for optimising uptake and security in online recruitment; procedural requirements for ensuring informed consent and data privacy in virtual interview settings; recommendations for supporting IT literacy for researchers and participants; discussions of call-recording functions for post-interview transcription purposes; and technical suggestions for maximising video-call quality (Lobe et al., 2020; Roberts et al., 2021; Topping et al., 2021). Understandably, given the urgency of these questions, literature written during the pandemic has not emphasised the longer-term methodological implications of the turn to the digital in qualitative interviewing. Yet, we can carry methodological questions raised before 2020 and lessons learned from the pandemic into a post-COVID-19 world (Davies et al., 2020; Kaufmann & Tzanetakis, 2020; Krouwel et al., 2019).

This paper argues that video interviewing should be considered less as an adaptive compromise within pandemic restrictions and more as an opportunity for longer-term methodological progress. While most discussions of recent technological adjustments to data collection are generalised, this paper focusses on specific methods. It scrutinises digital innovations: firstly of traditional methods, such as focus groups, and secondly of emergent semi-structured methods, such as the Grid Elaboration Method.

### Innovating Qualitative Methods

#### Updating traditional methods

Adaptations to longstanding qualitative methods during COVID-19 prompted beneficial updates to their flexibility and efficacy. Focus groups provide an instructive example. These had started to shift online before the pandemic (Brown et al., 2021), but were mostly text-based, utilising online messaging platforms and social media chat functions (Chen & Neo, 2019; Lijadi & van Schalkwyk, 2015). The literature has focussed on its drawbacks: excluding participants with less access to technology; promoting less detailed responses; precluding non-verbal cues such as body language; and sustaining less trust and engagement (Chen & Neo, 2019). There is some research suggesting strengths of conducting focus groups using video-calling technology (Tuttas, 2015), including similar richness of data to face-to-face comparisons (Daniels et al., 2019). Yet, discussion of digitalising traditional interviewing methods such as focus groups is still limited.

The pandemic provoked refreshed exploration of video interviewing, particularly for focus groups. Lobe et al. (2020) discussed their practical and ethical challenges and considerations during COVID-19: for example, recommending lower participant numbers online, given the difficulties of managing group dynamics remotely. They also proposed brief one-to-one pre-sessions with each participant to minimise the risk of unforeseen technological disruptions (Lobe et al., 2020). One empirical focus group study investigated UK experiences of COVID-19-related social distancing using video calling, but included little reflection on the methodology itself (Williams et al., 2020). The authors mentioned brief generalised strengths and weaknesses of online research, but overlooked particular implications of video interviewing – suggesting, perhaps, an implicit assumption that virtual adaptations are somewhat equivalent to face-to-face traditions.

Some, very recent studies, meanwhile, have contributed insights into the methodological advantages of conducting video-call focus groups. Two online video focus-group studies with low-socioeconomic-status African American participants yielded access to marginalised, underrepresented populations by facilitating travel-free contact with geographically dispersed participants (Lathen & Laestadius, 2021). These studies promoted reflection on the greater geographic, cultural and socioeconomic diversity of participants that online research can access. Prior discussions have lacked substantive consideration of equity issues. Despite noting limitations of working virtually – including variable participant familiarity with video-call platforms – the researchers described achieving safe, non-judgmental spaces for engaged, trusting conversations between participants. Focus groups can empower minority populations who might prefer sharing views among other participants from their community—and online focus groups may provide greater comfort for self-expression than unfamiliar settings.

Virtual-focus-group participants also reported enjoying having their voices heard and connecting with others during the pandemic (Lathen & Laestadius, 2021). In emergent virtual methods (below) too, participants have reported positive experiences, finding interviews “helpful” as “sounding boards” (Keen et al., 2021). Participant expressions of enjoyment and feeling heard are also prevalent in face-to-face interviews, suggesting commonality with digital adaptations. Especially during periods of impaired travel or social connectivity, being heard in virtual interviews may hold extra appeal for prospective participants, including those otherwise underrepresented in research.

Furthermore, technology-assisted transcription tools promise particular advantages for focus groups. Capturing precise dialogue requires verbatim transcription, yet determining which participant is speaking from group recordings containing overlapping voices is difficult (Tuttas, 2015). Video-calling technology innovations provide more sophisticated capacities for clarity in recording and transcribing. Video-call platforms include Zoom, Google Meet, Microsoft Teams and Skype. Built-in recording functions of video-call platforms allow researchers to record (with consent) both via audio and video. This facilitates clearer audiovisual records and may also support greater ecological validity without the intrusiveness of visible, physical cameras. Digitalising focus groups and other traditional qualitative methods thus yields distinct advantages, beyond COVID-19-related contexts.

#### Enhancing emergent methods

COVID-19 has also catalysed virtual evolutions of emergent qualitative methods, exemplified by the Grid Elaboration Method (GEM; Eiguren et al., 2021; Mondragon et al., 2021a; Mondragon et al., 2021b). The GEM is a free-associative data collection method devised by Joffe and Elsey (2014), employing psychodynamic principles of tapping latent cognitions and affects in participants’ spontaneous associations. It elicits participants’ first thoughts about a given research topic via their filling in 2x2 grids (Appendixes 1–2) and elaborating on their own associations in semi-structured interviews. Thematic analyses of these free-association-based interview responses have elucidated conceptualisations of diverse phenomena, ranging from city-dweller representations of strangers (Zeeb & Joffe, 2021) to loneliness (Fardghassemi & Joffe, 2021), and lay notions of neuroscience (O’Connor & Joffe, 2014). They have also revealed how people conceptualise risk-related phenomena, including climate change, natural disasters and emerging infectious diseases (Idoiaga Mondragon et al., 2017; Park & Mortell, 2020; van Soest et al., 2019).

The GEM exemplifies more specialised qualitative methods, where employing procedures in person appears preferable (Joffe & Elsey, 2014). Yet, it also exemplifies how such procedures can nonetheless flourish when transposed online. Three recent studies used the GEM remotely, to examine social representations of COVID-19 (Eiguren et al., 2021; Mondragon et al., 2021a, 2021b). Replacing researcher-participant interactions, they elicited grid responses and subsequent elaborations through online questionnaire platforms, inviting participants to fill in Google Form boxes. This adaptation circumvented difficulties of organising virtual meetings when the pandemic began, before video calling was popularised. Although potentially reducing social desirability effects by removing researcher presence, such written interviews sacrifice researchers’ ability to ensure in real time that participants’ elaborations are immediate and free associative, as the method emphasises.

Another study transposed the full GEM interview format online, through real-time video calls. It examined how people with chronic pain conceptualized it, inviting participants to fill in paper grids remotely (Keen et al., 2021). Participants could photograph their completed grid and email it to the researcher, or hold it to camera to be screenshotted by the researcher. Video calling – then in wider public use – enabled the researchers to encourage more immediate free-associative responses, and for participants to draw images (not just words).

These studies demonstrate how even complex, emergent methods could be productively digitalised during the pandemic. Their challenges and successes offer a lens for further exploring the methodological strengths of virtual interviews using the GEM and other qualitative methods. Several strengths are outlined below: namely, virtual methods’ accessibility and recruitment viability, scope for interview rapport, data richness and transcription efficiency.

#### Accessibility and recruitment

Firstly, virtual interviewing has widened geographic access to participants (Eiguren et al., 2021; Keen et al., 2021). Although home confinement during COVID-19 lockdowns may have increased openness to participation, the ease of video calls (rather than travelling) was likely itself a substantial incentive. Further, not travelling lends participants flexibility to fit interviews into tighter schedule gaps or reschedule at short notice. Also, while it used to be thought that digital data collection would bar older age groups from the research, increasing older-age familiarity with digital methods means that older participants are not significantly precluded from virtual interviewing (Vergouw et al., 2020).

Pre-interview logistics are also highly feasible online. This corroborates literature discussing virtual alternatives to gaining informed consent while preserving data confidentiality (Roberts et al., 2021; Sy et al., 2020). Participants have printed, signed, scanned and emailed consent forms, or simply e-signed them. This may deter those, particularly older, participants less familiar with technology – as in Keen et al. (2021) – yet the growing popularity of video calling may be increasing the comfort of older people with technological tasks.

#### Scope for interview rapport

Virtual interview interactions themselves have demonstrated significant emulation of natural conversation (Roberts et al., 2021). Early rapport is aided by warm, open on-camera researcher body language, thanking participants for their time, checking for any technical problems and reminding participants of their rights to pause or end the interview at any time – including pausing audio and visuals. Methods where researchers must view paper-based participant responses to prompt further questioning will require participants to photograph and email these responses, or hold them to camera to be screenshotted by the researcher. For semi-structured interviews requiring time-sensitive segments, time spent on different interview stages can be monitored on-screen more subtly than in person. This ability may aid rapport by reducing the risk of appearing distracted.

#### Data richness

Additionally, data elicited via video calls have demonstrated substantial richness akin to that gained in face-to-face contexts. Factors influencing the quality of the data collection, such as systematicity in the application of interview procedures and participant-researcher real-time rapport, can be fulfilled through virtual methods. Participant choice regarding the setting in which they are interviewed, provision of clear instructions and establishment of online rapport have facilitated extended elaborations for detailed thematic analyses (Keen et al., 2021; Roberts et al., 2021).

#### Transcription efficiency

Lastly, transcription of interviews can be supported by audio files captured directly from the video-call platform. This increases clarity of recordings, channelling audio input directly from participants’ electronic devices. Innovative transcription tools hold significant potential for improving researchers’ capacity to conduct interviews in varied formats and transcribe them reliably. Platforms including Zoom, Microsoft Teams and Skype provide automated transcription functions.

### Additional digital adjustments

Virtual research during COVID-19 has demonstrated that traditional and emergent qualitative methods can flourish remotely. Yet, this digitalisation can be further innovated. Across all virtual qualitative research methods, researchers and participants could turn off their cameras while participants are completing prompts to be used in later interview stages—for example, when filling in grids in the GEM. In work meetings, switched-off cameras may cause less engagement (Karl et al., 2021), but when invited explicitly in research contexts they may bring greater benefits. Researcher presence necessarily contaminates research contexts and may influence participant responses (Schwarz, 2007), yet digitalisation allows, in literal terms, participants to be alone in the room as they approach a given task. Participants’ not being on camera for certain parts of the research encounter may potentially lessen social desirability effects and enhance grounds for ecological validity. That said, there is still likely to be awareness that the researcher will see the data generated after the period in which cameras are switched off, and rapport can be further built once cameras are reactivated.

Moreover, in virtual group interviews, hand-raising functions embedded in video platforms can enhance how participants interact and share space in discussions. That said, group dynamics pervading face-to-face interviews should still be considered online: such as whether men may on average exercise greater confidence in hand-raising or speak for longer once called on (Krueger & Casey, 2014). Researchers should also ensure that participants who are more comfortable with technology do not dominate virtual group dynamics, such as by offering technical support before interviews to participants who self-report as less familiar with the video platform used. Enquiring about previous experience with video communication, providing technical support and establishing rules for virtual engagement may further facilitate equitable participation.

Secondly, video platforms such as Zoom and Microsoft Teams provide emerging options for real-time transcriptions displayed on screen. This may support researchers and participants to understand each other if either or both are speaking in non-native languages or have hearing impairments – more effectively than in person – by supplementing auditory information with text.

During COVID-19, interacting online became the setting for navigating stress, loneliness, and uncertainty of lockdowns. In this uniquely digitised context, being interviewed on video platforms may have felt increasingly familiar. It has opened new opportunities for accessibility and representation in research, and prompted valuable methodological innovations for further, post-pandemic research.

### Virtual Methods for Evolving Contexts

#### After COVID-19

While digitalising qualitative methods allowed research to function through global disruptions, it revealed a wider range of longer-term advantages. Recent discussions of virtual interviewing are still limited to coping strategies against social-distancing hindrances (Sy et al., 2020). Having shown above that video calling can widen access, increase ecological validity and enhance interview interactions within specific methodologies, this paper turns to discussing the implications of digitalising methods across different research contexts.

#### Supporting impaired mobility

In a pandemic, researchers and participants alike are prevented from travelling to face-to-face interviews by social distancing measures. Yet, at any time, some populations may have short- or long-term mobility difficulties. This includes people with specific physical disabilities, for whom video calls may be significantly more convenient than travelling to research institutions, hosting researchers, or visiting participants (Pegg et al., 2021; Williams et al., 2018). Some older-age participants might also prefer virtual contact than to travel to, or host, researchers, particularly given rising older-age familiarity with video-call platforms (Teo et al., 2019). Furthermore, populations with particular mental health difficulties such as panic disorder and agoraphobia might prefer conducting or participating in virtual interviews. Virtual interviews may also be more manageable for participants or researchers with caring responsibilities (Henderson & Moreau, 2020; Horrell et al., 2015). Moreover, people with chronic health conditions such as chronic pain or chronic fatigue may prefer to avoid travelling, or may feel more able to focus in online interviews. It also lends greater flexibility to these participants – who may not know how they will feel on the day – for committing provisionally to interview times and rescheduling at short notice. Future studies should harness the efficiency and compassion offered by virtual approaches to interviewing people with travel and mobility challenges.

#### Enhancing international research

Virtual interviewing is also promising regarding international research. COVID-19 has already prompted greater online communication between international research communities, increasing the collective efficacy of global collaborators (Meskell et al., 2021) and inclusivity of academic conferences, now virtual (Henderson & Moreau, 2020; Salomon & Feldman, 2020). Yet, beyond this pandemic, video-calling technology can empower faster, more affordable, more ethical international research. Connecting with participants virtually grants inexpensive, immediate access to global cultures, which researchers may not have financial or personal capacity to visit. This may particularly benefit research in regions affected by natural disasters or human conflict, to which travel has become dangerous or prohibited. Virtual methods may also empower studies in marginalised, displaced, or particularly secluded communities. Video-calling technology can enable local collaborators to visit secluded or deprived communities to recruit participants and support them to engage in video calls with non-local researchers. This bears significant ethical benefits. Firstly, it is important to draw on the community expertise of local teams when researching vulnerable and othered cultures (Mackenzie et al., 2007; Nordling, 2017; Pittaway et al., 2010), especially if researchers cannot visit in person (Roberts et al., 2021). Secondly, it addresses socioeconomic disparities in access to technology: local researchers can bridge technological gaps across locations and social groups, giving (video) platforms to marginalised voices. They can also bridge linguistic and cultural barriers to participation, acting as both introductory and retrospective translators. Moreover, elevating local voices within the research process may ameliorate potential power imbalances in traditionally Western-centric academic perspectives (Pittaway et al., 2010). Even as global travel is reallowed, virtual innovations may offer overall enhancement to international research ethics and efficacy.

#### Increasing theoretical validity

Beyond providing more ethical and equitable interview settings for numerous social groups and practical advantages for researchers, virtual innovations can also enhance qualitative research from a theoretical perspective. Video calls may allow employed participants to fit interviews more easily into their working days. Furthermore, allowing participants to engage from their homes grounds them in environments where everyday associations might not be disrupted by unfamiliar settings (Colom, 2021). This may lessen social desirability effects evoked by unfamiliar and potentially imposing institutional settings.

Use of virtual therapies is increasing, alongside increased evidence for their efficacy and desirability (Parikh & Huniewicz, 2015), and qualitative research can follow this example. Just as online therapy may reduce access barriers by normalising it within wider online interactions, participation in qualitative research can be normalised and widened through virtual contexts. The rise in virtual therapies has prompted nascent investigations into the mediating role of computers and other electronic devices within client-psychologist dynamics (Cataldo et al., 2021), and future methodological literature might further consider how device-mediated interview dynamics influence the data they elicit (Davies et al., 2020; Krouwel et al., 2019).

These theoretical benefits satisfy overarching principles for rigour, accountability and transparency within qualitative research (Gaskell & Bauer, 2000; Joffe, 2012). Digitalisation allowed emergent methods, otherwise unviable in the pandemic, to be used and developed. It also promises to reduce the costly and time-consuming aspects of qualitative research. As discussed, nascently before the pandemic, and accelerated during it, virtual interaction possibilities pave the way for further, novel research methods to be developed.

### Limitations of Virtual Research

These nascent modes of virtual research still carry limitations warranting consideration. Primarily, novel technologies require updated ethical scrutiny (Salmons, 2016). While some authors argue that most ethical concerns are the same as those found in face-to-face research (Lobe et al., 2020), others have raised concerns over data confidentiality and interview safety from cyber-interruption (Roberts et al., 2021; Sy et al., 2020). These issues have proven surmountable, as multiple video-call platforms have strengthened their security by introducing password-entry features (Yuan, 2020). Interview recordings can also be downloaded directly to researchers’ passworded computers and stored in folders disconnected from the internet. However, researchers should ensure, for instance, that automatic transcription functions do not transmit data to cloud servers.

There are also some methodological drawbacks. Using automatic transcription may render some researchers less familiar with their data than if transcribing manually, where deciphering interviews supports researchers to absorb their content. However, manual transcription may soon become outdated, given the numerous automated transcription applications already available. Research projects have also historically hired transcribers, so researchers are usually not embedded in their data at the transcription stage anyway. Also, counter to the strengths raised above, since researchers do not visit in person the contexts under investigation, they should embed themselves in the culture in alternative ways, such as exploring regions using wider literature, news media, talks and even interactive virtual maps (Roberts et al., 2021). Additionally, video interviewing participants in their home environments risks susceptibility to distractions: from childcare and pets to work-email notifications (Rahman et al., 2021). Some participants have also reported distraction from seeing themselves on-screen (Deakin & Wakefield, 2013; Seitz, 2016), so virtual researchers could invite interviewees to disable this feature. Moreover, video-calling can fatigue participants faster (Epstein, 2020), so interview length should be limited. It is also challenging to support participants remotely if they experience emotional distress during interviews. Lastly, all virtual research must consider how to correct socioeconomic, age-related and individual disparities in access and openness to computer, internet and video-calling technology.

### Comparing Virtual and Face-to-Face Qualitative Research

Virtual interviewing presents multiple commonalities with face-to-face methods, supporting its methodological legitimacy. It also displays key differences – both improvements and limitations – which merit concise delineation.

Advertisement, recruitment and interview organisation are largely online in both cases. Core interviewing approaches – from semi-structured interviews to specialised procedures – remain unchanged online. Facial and vocal cues are retained in video calls, and rapport can be developed. Participants express enjoyment and feeling heard in both settings. Focus group dynamics are analogously pervasive online. Both settings accommodate interview recording.

However, virtual interviewing can exclude communities and individuals without technological competence or access. Consent is more difficult to obtain, and data confidentiality requires caution. Body language is less observable, and participants may fatigue faster or face home distractions. It is harder to view participants’ written prompts in specialised methods requiring them.

Yet, virtual interviewing provides enhanced recording capacities, with clearer audio capture and automatic transcription, including real-time captioning. It offers instant messaging and screen-sharing options, and recordings can be video as well as audio. Interview scheduling is more flexible, and travel expenses are removed. It enables access to remote communities, regions where travel is prohibited and participants with impaired mobility. It also supports greater ecological validity, removing institutional settings and reducing researcher presence.

**Figure 1.**
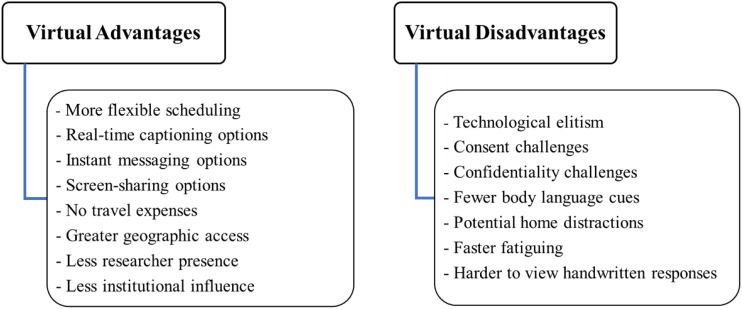
Virtual interviewing: advantages and disadvantages.

**Figure 2.**
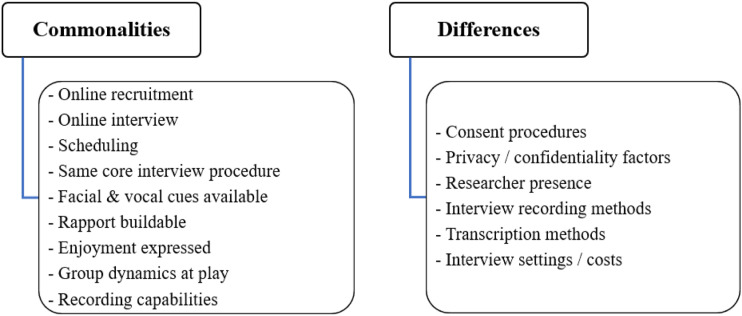
Virtual versus face-to-face interviewing: commonalities and differences.

Figure 1 summarises these advantages and disadvantages of virtual interviewing. Figure 2 summarises the commonalities and differences between virtual and face-to-face interviews.

## Conclusions

Research communities around the world have rallied to support global efforts to understand and overcome the COVID-19 crisis. These concerted efforts have contributed significant innovation in research methods to surmount unprecedented barriers. Such innovation need not be lost when these barriers subside. The pandemic has encouraged increased global interest in virtual qualitative research methodologies already emergent in the literature: particularly, video interviewing.

Yet, discourse surrounding this innovation has retained a tone of compromise. This paper seeks to shift the emphasis from coping to opportunity and progress. Growing evidence from cutting-edge research suggests that virtual interviewing can thrive in its own right. Traditional methods such as focus groups and emergent methods including the GEM can find refreshed efficacy in virtual formats. As virtual studies proliferate across varied research contexts, they pose unique promise for greater access, efficiency and ethicality.

In the same way that virtual therapy has not supplanted face-to-face therapy conventions, so digital research need not supplant in-person research conventions; it can complement them and simultaneously yield wider access (Parikh & Huniewicz, 2015). Traditional research approaches have historically taken place in face-to-face settings. However, in an increasingly digital world, pressures of social distancing have forced known methods to adapt. An opportunity for innovation has arisen from this challenge, and as we emerge from the pandemic and look to future research, this paper has argued why virtual methods should retain a central place.

## Appendix 1 GEM Instructions (Keen et al., 2021)

We are interested in how you understand chronic pain – that is, how chronic pain works, why it starts and carries on. Please list the different images and words you associate with your chronic pain using these boxes. Include everything you associate with one image and/or word into one box. Don’t worry how good an artist you are, a really simple drawing can be a great way of portraying your thoughts and feelings. You can use any word more than once in different boxes.
